# Induction of Tachykinin Production in Airway Epithelia in Response to Viral Infection

**DOI:** 10.1371/journal.pone.0001673

**Published:** 2008-03-05

**Authors:** James P. Stewart, Anja Kipar, Helen Cox, Catherine Payne, Sylvia Vasiliou, John P. Quinn

**Affiliations:** 1 Department of Medical Microbiology, The University of Liverpool, Liverpool, United Kingdom; 2 Department of Veterinary Pathology, The University of Liverpool, Liverpool, United Kingdom; 3 Divisions of Physiology & Human Anatomy Cell Biology, The University of Liverpool, Liverpool, United Kingdom; 4 Scottish Centre for Regenerative Medicine, University of Edinburgh, Edinburgh, United Kingdom; Emory University, United States of America

## Abstract

**Background:**

The tachykinins are implicated in neurogenic inflammation and the neuropeptide substance P in particular has been shown to be a proinflammatory mediator. A role for the tachykinins in host response to lung challenge has been previously demonstrated but has been focused predominantly on the release of the tachykinins from nerves innervating the lung. We have previously demonstrated the most dramatic phenotype described for the substance P encoding gene preprotachykinin-A (*PPT-A*) to date in controlling the host immune response to the murine gammaherpesvirus 68, in the lung.

**Methodology/Principal Findings:**

In this study we have utilised transgenic mice engineered to co-ordinately express the beta-galactosidase marker gene along with *PPT-A* to facilitate the tracking of *PPT-A* expression. Using a combination of these mice and conventional immunohistology we now demonstrate that *PPT-A* gene expression and substance P peptide are induced in cells of the respiratory tract including tracheal, bronchiolar and alveolar epithelial cells and macrophages after viral infection. This induction was observed 24h post infection, prior to observable inflammation and the expression of pro-inflammatory chemokines in this model. Induced expression of the *PPT-A* gene and peptide persisted in the lower respiratory tract through day 7 post infection.

**Conclusions/Significance:**

Non-neuronal *PPT-A* expression early after infection may have important clinical implications for the progression or management of lung disease or infection aside from the well characterised later involvement of the tachykinins during the inflammatory response.

## Introduction

The tachykinin family of neurotransmitters are not only involved in central and peripheral nervous system function but also have a role in inflammation (termed neurogenic inflammation) and adaptive immunity [1], [2]. The most characterized member of the family is substance P (SP). This is encoded by the preprotachykinin A (*PPT-A*) locus. Alternative splicing of *PPT-A* RNA and processing of the propeptide yields, in addition to SP, neurokinin A (NKA) and neuropeptides K and γ. Individual spliced forms invariably encode SP and a variable combination of these other peptides. A major physiological source of SP is primary sensory neurons, whose cell bodies in the dorsal root ganglia produce SP and transport it to peripheral and central sites where it is stored and later released from nerve endings. Although local nerves have been believed to be the major source of tachykinins in the peripheral tissues, *PPT-A* has been shown to be induced and expressed in other cell types such as monocytes, macrophages, pancreatic islet cells and various tumour cell types [3]–[7]. This has led to the hypothesis that SP not only acts as a mediator of the neuroimmune system but is also involved in direct interaction between immune cells in a paracrine and/or autocrine fashion independent of sensory nerves [4], [8]. The tachykinins can modulate the immune response and SP has been shown to regulate production of a number of cytokines including IL-1, IL-6, IL-8 and TNFα to mediate inflammatory and cell proliferative responses [9], [10]. In addition to the classical peptides SP and NKA, the recently discovered peptides such as hemokinin 1 and the endokinins have been found in non-neuronal cells/tissues such as pulmonary and cardiovascular tissue, articular cartilage and cells of the immune system [11], [12] and they have high affinity SP receptors, NK-1^R^ [13] adding a further layer of complexity to the tachykinin function in host defence. It is therefore important to define the temporal and spatial regulation of the tachykinins and hemokinins in the periphery in response to challenge. The generation of transgenic mice that co express the *PPT-A* gene with the *LacZ* marker gene [14], [15] has allowed us to explore the expression and function of the *PPT-A* gene in this manner in the lung.

The tachykinins have been extensively implicated in the initiation and progression of lung disease processes such as bronchitis and asthma [16]–[19]. It has been suggested that SP, NKA and their respective high affinity receptors NK-1^R^ and NK-2^R^ are important. These peptides would have different functions in the lung as they contract smooth muscle cells mainly by interaction of NKA with NK-2^R^, while an increase in vascular permeability and pro-inflammatory effects are mediated by SP on the NK-1^R^ [16]. In such a manner tachykinins are potent contractors of airways producing a dose-related bronchoconstriction when administered by means of inhalation to asthmatic subjects [20]. Similarly a number of studies have shown that viruses (e.g. respiratory syncytial virus and murine gammaherpesvirus, MHV-68) can induce SP and neurogenic inflammation, particularly in lungs in the context of a respiratory challenge [21]–[23]. In addition, NK1 is upregulated in lymphocytes (especially T cells) in the lung in response to viral infection in the lung [24] and antibody to SP decreases inflammatory responses to pulmonary viral infection [25]. This indicates a primary role for SP in the lung in the response to respiratory viral infection. These actions of the tachykinins in response to virus infection are similar to bronchitis and asthma [21], [22], [26], [27]. We demonstrate here that induction of the *PPT-A* gene and the subsequent synthesis of SP in specific non neuronal cell populations in the lung are an early response to MHV-68 infection. This further supports a model where induction is initiated by interaction of the cell with the virus particle.

## Results

### PPT-A gene expression is induced in airways following MHV-68 infection

Tachykinins, including SP are found in increased levels in the lung after respiratory infection. However, the exact source of the tachykinin peptides has never been precisely identified. To determine if tachykinin gene expression was induced locally in airways in response to infection with a respiratory virus, we initially utilised transgenic mice (143-YAChPPT-ALacZ) that had been engineered to co-ordinately express a transgenic, human copy of *PPT-A* (*htPPT-A*) and the *LacZ* reporter gene. The product of the latter can readily be rapidly and easily detected in tissues by a simple staining method. Thus, LacZ staining in these mice is a surrogate marker for *htPPT-A* gene expression. 143-YAChPPT-ALacZ mice were infected intranasally with murine gammaherpesvirus 68 (MHV-68) and groups of 3 mice were euthanized at 1, 3 and 7 days post-infection. Lungs, including trachea and lymph nodes were removed and stained whole using X-gal as substrate. Mock-infected mice served as controls.

**Figure 1:**
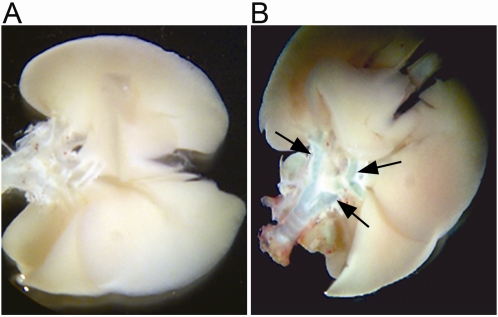
LacZ expression in lungs from infected and uninfected 143-YAChPPT-ALacZ mice. Groups of 143-YAChPPT-ALacZ mice were infected or mock-infected with MHV-68 and euthanized at various time-points post-infection. Micrographs show whole lung blocks from mock-infected (Panel A) and infected (Panel B) mice at 1 day p.i. stained for the presence of the LacZ marker (blue). Staining for LacZ is observed within trachea and bronchi (arrows) of the infected mouse only

The results (Fig. 1) showed that there was no detectable LacZ staining in mock-infected mice at any time-point (Panel A). However, as early as day 1 p.i., LacZ staining was observed in airways of lungs from infected mice (Panel B). This staining persisted through day 7 p.i.. *htPPT-A* gene expression in the context of the well characterised transgenic model was therefore induced locally in airways after MHV-68 infection.

### Epithelial cells and macrophages in airways are induced to express *PPT-A* during infection

To identify precisely the cell types induced to express *PPT-A* in airways, groups of 143-YAChPPT-ALacZ mice were infected with MHV-68 as above and euthanized at 1, 2 , 3 and 7 days p.i. Mock-infected mice served as controls. Lung tissue including trachea and lymph nodes was snap-frozen and cryosections prepared. Sections were stained for the presence of LacZ and examined by light microscopy. HE-stained sections served to assess any morphological alterations.

**Figure 2:**
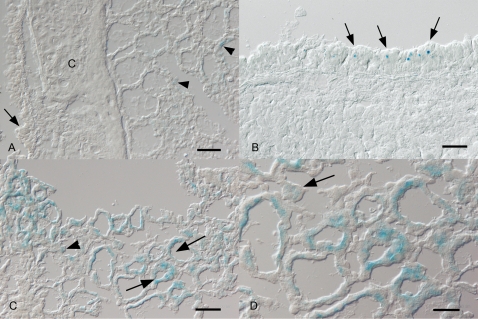
Histological detection of PPT-A expression in respiratory tissue. Groups of 143-YAChPPT-ALacZ mice were infected or mock-infected with MHV-68 and euthanized at various time-points post-infection. Micrographs show trachea and lung tissue sections stained for the presence of the LacZ marker (blue; frozen sections). A. Mock-infected 143-YAChPPT-ALacZ mouse. LacZ expression is observed in individual bronchial (arrow) and alveolar (arrowheads) epithelial cells. C: bronchial cartilage. Bar = 40 µm. B. 143-YAChPPT-ALacZ mouse, day 1 post infection. A large patch of tracheal epithelial cells exhibit LacZ staining (arrows). Bar = 20 µm. C. 143-YAChPPT-ALacZ mouse, day 1 post infection. Several alveoli exhibit LacZ staining of all epithelial cells (arrows), in others only occasional cells, often with the morphology of type II alveolar epithelial cells (arrowhead) are positive. Bar = 40 µm. D. 143-YAChPPT-ALacZ mouse, day 1 post infection. Higher magnification of Panel C, showing staining for LacZ in numerous alveolar epithelial cells. A positive syncytial cell is also seen (arrow). Bar = 10 µm.

The results (Fig. 2) showed LacZ staining in occasional alveolar and bronchial epithelial cells in mock-infected mice (Panel A). As early as day 1 p.i. with MHV-68 there was a general increase in the numbers of LacZ-positive tracheal, bronchial and alveolar epithelial cells. Trachea and bronchi often exhibited patches of positive epithelial cells (Panel B). The staining in the lung parenchyma was also patchy. Some alveoli contained single, scattered positive epithelial cells, mainly with the morphology of type II pneumocytes, others were entirely positive (Panels C, D). Occasionally, positive desquamated alveolar epithelial cells/macrophages as well as positive syncytial cells were seen in alveolar lumina (Panel D). On days 2, 3 and 7 p.i., staining was similar, but appeared gradually less extensive than on day 1 p.i. This was obvious in a lower number of alveoli exhibiting positive cells. However, entire alveoli with positive cells were found at each time point.

These results indicate that a small number of cells in the respiratory tract constitutively express the *htPPT-A* transgene in uninfected mice. However, a large number of airway epithelial cells and macrophages are rapidly induced to express the *htPPT-A* gene after respiratory challenge with MHV-68.

### Substance P peptide expression is induced along with *PPT-A* gene expression

**Figure 3:**
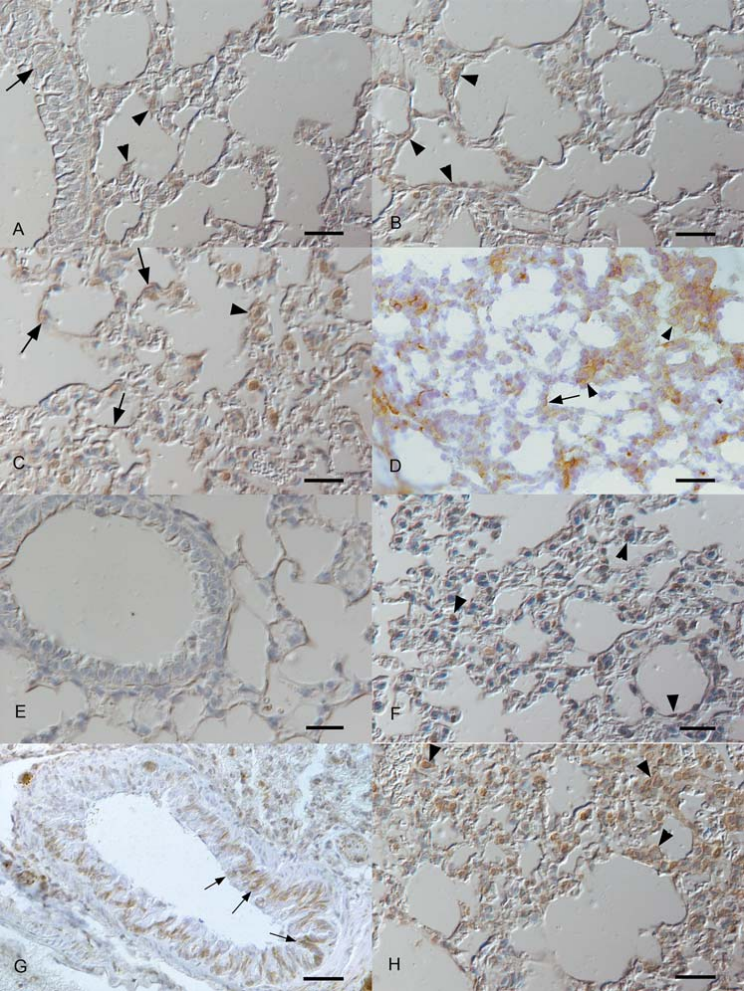
Immunohistological detection of SP expression in respiratory tissue. Groups of 143-YAChPPT-ALacZ mice (Panels A-E) and BALB/c mice (Panels F-H) were infected with MHV-68 or mock-infected and euthanized at various time-points post-infection. Micrographs show airway and lung tissue sections stained for the presence of SP (brown; frozen or paraffin-embedded tissue sections, peroxidase anti-peroxidase method, anti-SP, Papanicolaou's haematoxylin counterstain). A, B. Mock-infected 143-YAChPPT-ALacZ mouse. SP expression is seen as very weak cytoplasmic staining of occasional bronchial epithelial cells (arrow) or alveolar epithelial cells (arrowheads). Bars = 20 µm. C, D. 143-YAChPPT-ALacZ mouse, day 1 post infection. Numerous alveolar epithelial cells stain positive for SP (arrows); some of these can be identified as type II pneumocytes (arrowheads). C: PFA-fixed and paraffin wax-embedded tissue section, D: Frozen section. Bars = 20 µm. E. 143-YAChPPT-ALacZ mouse, day 1 post infection. No reaction is seen in the negative control section, where the primary antibody was replaced by tris-buffered saline. Bar = 20 µm. F. Mock-infected BALB/c mouse. SP expression is seen as cytoplasmic staining of occasional alveolar epithelial cells (arrowheads). Bar = 20 µm. G. BALB/c mouse, day 2 post infection. Numerous bronchial respiratory epithelial cells exhibit moderate to intense staining for SP (arrows). Bar = 20 µm. H. BALB/c mouse, day 1 post infection. Numerous alveolar epithelial cells stain positive for SP; many of these can be identified as type II pneumocytes (arrowheads). Bar = 20 µm.

To confirm that SP peptide was being produced along with *PPT-A* gene expression, frozen sections consecutive to those used for LacZ staining and sections prepared from paraformaldehyde (PFA)-fixed, paraffin wax-embedded tissue from the same mice analyzed above were immunohistologically stained for SP. The results are shown in Fig. 3.

In mock-infected mice, SP expression was observed in occasional alveolar and bronchial epithelial cells (Panel A, B). Some positive alveolar epithelial cells had the morphology of type II pneumocytes. Desquamated alveolar macrophages, if present, were intensely positive.

On day 1 p.i., mice exhibited numerous individual and patches of tracheal and bronchial epithelial cells which stained positive for SP. In alveoli, positive epithelial cells were seen, some of which had the morphology of type II pneumocytes (Panels C, D). Positive desquamated epithelial cells and macrophages were occasionally observed within alveolar lumina. On days 2, 3 and 7 p.i., airway epithelial expression patterns were similar to those seen at day 1 p.i., with similar numbers of positive cells on days 2 and 3, but overall lower numbers of positive cells on day 7 p.i.

No reaction was seen in a negative control where the primary antibody was replaced with buffer (Panel E).

Thus, SP expression is induced after infection, co-ordinate with *htPPT-A* gene expression as both are detected in similar cells in trachea, bronchi and alveoli, and with a similar focal distribution.

### Induction of SP expression occurs in non-transgenic mice

Although previous studies have shown that the pattern of LacZ staining corresponds to known endogenous (non-transgenic) *PPT-A* gene expression in mice, we were concerned that there might be differences between expression in the 143-YAChPPT-ALacZ mice and non-transgenic mice. We therefore repeated the above experiments using BALB/c mice and stained PFA-fixed, paraffin wax-embedded tissue sections for SP expression.

The results (Fig. 3 F–H) showed that the pattern of SP staining in BALB/c mice was comparable to that seen in 143-YAChPPT-ALacZ mice. Thus, SP expression was observed in occasional, scattered bronchiolar and alveolar epithelial cells in uninfected mice (Panel F). In infected mice, there were numerous positive tracheal, bronchial and alveolar epithelial cells and alveolar macrophages (Panels G, H). The numbers of positive cells appeared again greatest at days 1–3 p.i..

Thus, the induction of SP expression in airways after infection was also validated for the endogenous murine *PPT-A* gene.

### Foci of *PPT-A*/SP expression correspond with areas of viral infection

**Figure 4:**
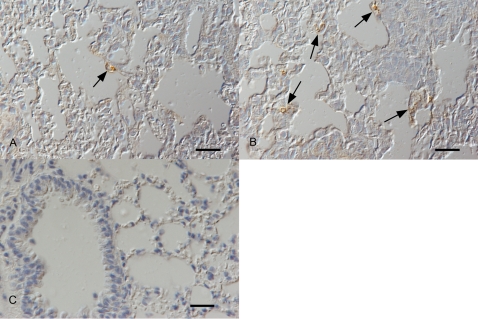
Immunohistological detection of MHV-68 antigen in respiratory tissue. Groups of BALB/c mice were infected with MHV-68 and euthanized at various time-points post-infection. Micrographs show airway and lung tissue sections stained for the presence of MHV-68 antigen ((brown; paraffin-embedded tissue sections, peroxidase anti-peroxidase method, anti-MHV-68, Papanicolaou's haematoxylin counterstain). A. BALB/c mouse, day 3 post infection. MHV-68 antigen is expressed by an individual type II alveolar epithelial cells (arrow). Bar = 20 µm. B. BALB/c mouse, day 7 post infection. MHV-68 antigen is expressed by several individual and small groups of alveolar epithelial cells (arrows). Bar = 20 µm. C. BALB/c mouse, day 7 post infection. No reaction is seen in the negative control section, where the primary antibody was replaced by tris-buffered saline. Bar = 20 µm.

During the acute phase of infection, MHV-68 predominantly infects airway epithelial cells, in particular alveolar epithelial cells. To determine whether the foci of viral infection corresponded to the distribution of SP expression after infection, serial sections were cut from the lung tissue of the infected 143-YAChPPT-ALacZ and BALB/c mice and stained for either LacZ/SP or MHV-68 antigen. Due to the kinetics of MHV-68 replication and spread in the host, viral antigen was not detected until day 3 p.i. when scattered individual alveolar epithelial cells were positive (Fig. 4A). Viral antigen expression was more abundantly observed on day 7 p.i. (Fig. 4B). However, LacZ staining and MHV-68 antigen were observed in a similar alveolar epithelial location (Fig. 4A, B). No reaction was observed in a negative control where primary antibody was replaced by buffer (Panel C).

## Discussion

Sensory C-type fibres are thought to provide the predominant source of tachykinins, including SP, in the lung and have a role in the pathophysiological response to challenge in the lungs which have been well documented. However, our manuscript provides strong evidence that as a response to infectious challenge, tachykinin production in the lung is initiated locally in non-neuronal cells. This local tachykinin release may then contribute to the inititation of a cascade that includes the host immune response and the neurogenic inflammatory response.

To examine whether SP was being produced *in situ* in the lung as opposed to being released from nerve terminals, animals from a previously established transgenic mouse line expressing human *PPT-A* co-ordinately with the LacZ marker gene were used for the viral infections. In this transgenic line (143-YAChPPT-ALacZ), a YAC (yeast artificial chromosome) containing the human *PPT-A* locus had been isolated from a human cDNA library and an internal ribosomal entry site (IRES)-LacZ marker gene cassette had been cloned into the non-coding exon 7 through homologous recombination. LacZ expression is therefore a surrogate marker for *PPT-A* gene transcription and positive cells can be easily identified through X-gal staining for LacZ protein. Our previous analyses of the transgenic lines produced showed that the LacZ and *PPT-A* genes were being correctly anatomically expressed in the central and peripheral nervous system [15]. Although the respiratory tract has an extensive network of peptidergic innervation, in this study, epithelial cells lining the air passages of 143-YAChPPT-ALacZ mice were found to be transcribing *htPPT-A* mRNA in response to viral infection, as shown by the presence of blue staining in the airways corresponding to expression of the marker gene LacZ. Given that these animals produced the expected patterns of the endogenous *PPT-A* gene in the rodent nervous system we have no reason to believe that the use of the human allele would have inappropriate expression in the lung. However, we confirmed synthesis of SP protein in these cells by immunostaining for SP in both transgenic and non-transgenic (BALB/c) animals. In the non-transgenic animals, the SP produced can only be synthesised by the mouse gene. Further, we saw a similar pattern of SP-specific staining in the two mouse strains, indicating that the induction is not a strain specific response.

We have shown production of SP peptide by airway epithelial cells. *PPT-A* precursor protein needs to be processed into its constituent peptides by neutral endopeptidase (NEP; EC 3.4.24.11, also called CD10) before it becomes active. One concern therefore was that the nascent tachykinin peptide produced by epithelial cells might not be processed into active protein. However, it has previously been shown that NEP/CD10 is constitutively expressed by alveolar and airway epithelial cells *in vivo* [28]. This indicates that the PPT-A precursor observed in airway epithelia will be processed into active peptides and be able to exert their effects locally.

The *htPPT-A* transgenic model which co-ordinately expresses the LacZ marker allowed us not only to determine which cells were expressing *PPT-A* in response to challenge but also permitted us to determine the temporal cascade of expression. Expression of LacZ (and hence *htPPT-A*) in airway epithelial cells was initiated at a very early time after infection (24 h p.i.) and persisted, through 7 days p.i.. This is unusual as the induction of most pro-inflammatory and immunomodulatory mediators (e.g. IFN-γ, IL-6, IL-10, RANTES, MIP-1α, MIP-1β, IP-10) in the lungs of mice in viral respiratory challenge models does not occur until at least 3 days p.i., peaking around 6 through 10 days p.i. [29]–[32]. The timing of the *htPPT-A* and SP induction in airway epithelia is clearly earlier than this and more akin to that of IFN-α, which is rapidly induced in the lung during the first few days after infection [33]. The timing of SP induction is extremely significant and indicates that locally-produced SP is likely to be involved in the initiation of the host inflammatory and immune response in the lung, rather than being downstream of the induction of other inflammatory mediators. The effects of locally-produced SP are likely to involve paracrine and autocrine loops as human bronchial epithelial cell lines also respond to SP with a rise in synthesis and release of inflammatory cytokines in a receptor mediated fashion [34]. This is especially important for respiratory infection as the epithelial cells lining the lumen are the first to encounter and respond to invasion by pathogens.

An important role for tachykinins released from the non neuronal cells to infection has not only been demonstrated in our previous work [23] but also for respiratory syncitial virus (RSV). Consistent with this RSV, a respiratory viral infection similar to MHV-68, initiates a lower inflammatory response if the pulmonary neuronal SP release is inhibited [21] analogous in part to the response we had previously observed with MHV-68 [23]. In other organs a similar role for tachykinins synthesised in non neuronal cells has also been postulated. In murine *Schistosoma mansoni* infection, for example, ova embed in the liver and induce a local Th2-type granulomatous inflammatory response. SP is necessary for a normal immune response to this pathogen as shown by infection of NK1-R knock out mice [35]. We have also demonstrated that inappropriate expression of *PPT-A* and expression of SP in human chondrocytes is correlated with the progression of arthritis and the control of IL-4 expression in that model [36]. Together this set of data suggests stimulus inducible expression of the tachykinins in non neuronal cells may be a common response mechanism not only in the lung but in a variety of other cells.

An important and early role for tachykinins in the induction of inflammation and the immune response suggests that the selective use of tachykinin agonists or antagonists could be of use in modulating the response to pathogens. Agonists could enhance the response to pathogens or vaccines to improve clearance of a persistent virus or response to vaccination. In contrast, tachykinin antagonists might be of use where immunopathological processes resulting from infection cause morbidity or mortality (e.g. RSV infection in infants).

## Materials and Methods

### Infection of Mice

All animal work was performed under UK Home Office Project Licence number 40/2483 and Personal Licence number 60/6501. BALB/c mice were purchased from Bantin and Kingman (Hull, UK). The transgenic mouse line termed 143-YAChPPT-ALacZ containing the human Preprotachykinin-A (*PPT-A*) gene co-expressing the β-galactosidase (LacZ) reporter gene was produced by using a yeast artificial chromosome construct [14]. The expression of the human gene can be followed *in vivo* by LacZ staining.

Groups of animals were anesthetized with isoflurane and inoculated with 4×10^5^ PFU MHV-68 in 40 µl of sterile phosphate buffered saline (PBS). At various times post infection (p.i.), animals were euthanized and entire lungs with trachea and lymph nodes were harvested. Four mice from each group were euthanized at day 1 (24 h) p.i. and 3 animals from each group were euthanized at days 2, 3 and 7 p.i., respectively. Uninfected mice from each group served as controls.

### Detection of LacZ in entire lungs

Entire lungs with trachea and lymph nodes were rinsed and placed in solution A (1M PBS containing 1M MgCl_2_) for 30 mins at room temperature. They were then rinsed with solution B (1M PBS containing 1M MgCl_2_, 0.01% sodium deoxycholic acid, 0.02% IGEPAL) and placed in solution B for 5 mins at room temperature. Tissues were then stained by incubation for 20–24h at 37°C with staining solution (Xgal (1mg/ml) dissolved in 1M PBS containing 1M MgCl_2_, 0.01% sodium deoxycholic acid, 0.02% IGEPAL, 5mM potassium ferricyanide, 5mM potassium ferrocyanide). Staining solution was then removed and replaced with 1M PBS until photographs were taken on a dissecting microscope (not longer than two days after staining).

### Detection of LacZ in tissue sections

Lung tissue including trachea and bronchial lymph nodes were taken from 143-YAChPPT-ALacZ mice and snap-frozen at −80°C after embedding in Tissue Tek® (O.C.T.™ compound; Sakura, Zoeterwoude, The Netherlands). Sections (5–6 µm) were prepared on a cryotome and were stained with haematoxylin-eosin (HE), were used for the detection of *PPT-A* transcription by the staning for LacZ as above or were used for the immunohistological detection of substance P and MHV-68 antigen.

### Histology and Immunohistology

Additional lung samples were fixed in 4% buffered paraformaldehyde (PFA; pH 7.4) and were routinely embedded into paraffin wax. Sections (3–5 µm) were stained with HE or used for the immunohistological detection of SP and MHV-68 antigen.

For the detection of SP and MHV-68 antigen, the peroxidase anti-peroxidase (PAP) method was used. Briefly, paraffin-embedded sections were dewaxed in xylene and rehydrated through graded alcohol while Tissue Tek®-embedded frozen sections were rinsed in tris buffered saline (TBS; pH 7.6). After inactivation of endogenous peroxidase with 0.5% H_2_O_2_ in methanol, antigen retrieval by incubation with 10 mM citrate buffer (pH 6.0) at 97°C was performed on the PFA-fixed tissue sections. Slides were then incubated for 15–18 h at 4°C with the primary antibodies (rabbit anti-SP; BP823, DPC Biermann GmbH, Hiddenhausen, Germany; diluted 1∶100; rabbit anti-MHV-68, diluted 1∶2000) in TBS with 20% swine serum. Binding was demonstrated by the PAP method and visualisation with diaminobenzidin tetrahydrochloride. Consecutive negative control sections where the primary antibody was replaced by TBS were prepared for each section stained for SP or MHV-68 antigen.
